# Prognosis value of dynamic variation of tissue oxygen saturation during severe cardiogenic shock

**DOI:** 10.1186/cc9457

**Published:** 2011-03-11

**Authors:** P Gaudard, J Eliet, O Attard, P Colson

**Affiliations:** 1 CHRU, Montpellier, France

## Introduction

To evaluate the prognosis value of dynamic thenar O_2 _saturation (StO_2_) response using a vascular occlusion test (VOT) during cardiogenic shock.

## Methods

A retrospective clinical observational analysis was performed on adult patients treated for severe cardiogenic shock in a surgical ICU. The non-invasive InSpectra near-infrared spectrometer was used to assess the effect of VOT on thenar eminence StO_2_. The VOT manoeuvre was repeated within the first 24 hours of admission. StO_2 _VOT-induced changes were compared between surviving and nonsurviving patients between the first 8 hours and the next 16 hours.

## Results

**Figure 1:**
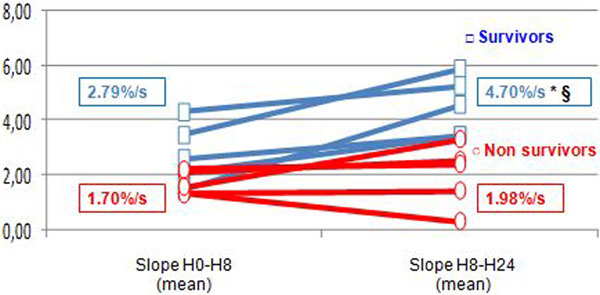
**StO_2 _recovery slope (mean)**.

Ten patients suffering from cardiogenic shock (age 59.8 ± 13.8 years; APACHE score 21.3 ± 5.9) were treated with inotropes (*n *= 7) and/or circulatory mechanical assistance (four IABP, three ELS, one LVAD) and vasopressors (*n *= 9). Mortality in the ICU was 50%. Hemodynamic and metabolic parameters were not different between survivors and nonsurvivors (Table 1). The post-VOT StO_2 _recovery slope tended to be faster within the first 8 hours in survivors than in nonsurvivors (2.8 ± 1.1 vs. 1.7 ± 0.4%/s, *P *= 0.09) and improved significantly in the H8 to H24 period (4.5 ± 1.2 vs. 2 ± 1.1%/s, *P *= 0.007). The post-VOT StO_2 _recovery slope increased significantly within the first 24 hours in all survivors (Figure 1).

**Table 1:** Hemodynamic parameters within the first 8 hours in the ICU

	Survivors	Nonsurvivors	*P *value
MAP	85	70	0.08
CI	2.4	2.3	1
ScvO_2_	65	57	0.45
Lactate	4.4	8	0.47
StO_2_	77	81	0.35

## Conclusions

Our results suggest that, in patients treated for cardiogenic shock, rapid improvement in the post-VOT StO_2 _recovery slope is associated with a better prognosis.

